# REDUCING HEALTH DISPARITIES IN THE ERA OF VALUE-BASED CARE

**DOI:** 10.1093/geroni/igz038.2080

**Published:** 2019-11-08

**Authors:** Amit Kumar, Elham Mahmoudi, Maricruz Rivera-Hernandez

**Affiliations:** 1 Northern Arizona University, Flagstaff, Arizona, United States; 2 University of Michigan, Department of Family Medicine, Ann Arbor, Michigan, United States; 3 Brown University, Providence, Rhode Island, United States

## Abstract

The US health care system is at a critical moment of transformation. The implementation of value-based models has made significant progress towards improving care quality and coordination, continuity of care and reducing cost. However, concerns have been raised regarding “cherry-picking” healthier people that may negatively impact patients with more complex needs and minority populations. Given that the US is becoming more diverse, there is a need for understanding the impact of social risk factors including ethnicity, immigration status, income and geography on health outcomes and issues of health care disparities. This panel brings together four studies that examine these phenomena in minority populations. These studies will provide novel insight regarding 1) healthcare utilization in Mexican-American Medicare beneficiaries and showing that social determinants of health are associated with a higher risk of hospitalization, emergency room admissions, and outpatient visits. 2) Mortality rates and predialysis care among Hispanics in the US, Hispanics in Puerto Rico, and Whites in the US demonstrating substantial disparities in access to recommended nephrology care for Hispanics in Puerto Rico; 3) Trends in age-adjusted mortality rates and supply of physicians in states with different nurse-practitioners regulation. 4) The impact of social risk factors on disenrollment from Fee-For-Service and enrollment in a Medicare Advantage plan in older Mexican-Americans. 5) Racial disparities in access to physician visits, prescription drugs, and healthcare spending among older adults with cognitive limitation. Studies in this panel will also discuss the effects of changes in care delivery and payment innovations in improving health equity.

